# Development of a complex intervention to improve mobility and participation of older people with vertigo, dizziness and balance disorders in primary care: a mixed methods study

**DOI:** 10.1186/s12875-021-01441-9

**Published:** 2021-05-12

**Authors:** Verena Regauer, Eva Seckler, Eva Grill, Richard Ippisch, Klaus Jahn, Petra Bauer, Martin Müller

**Affiliations:** 1 Centre for Research, Development and Technology Transfer, Rosenheim Technical University of Applied Sciences, Hochschulstraße 1, 83024 Rosenheim, Germany; 2 Institute for Medical Information Processing, Biometry and Epidemiology, Ludwig Maximilian University of Munich, Marchioninistraße 17, 81377 Munich, Germany; 3 German Centre for Vertigo and Balance Disorders, Ludwig-Maximilian University of Munich, Marchioninistrasse 15, 81377 Munich, Germany; 4 Practice Centre Neurology, Psychiatry and Psychotherapy Germering, Josef-Kistler-Straße 10, 82110 Germering, Germany; 5 Schoen Clinic Bad Aibling, Kolbermoorer Strasse 72, 83043 Bad Aibling, Germany; 6 Faculty for Applied Health and Social Sciences, Development and Technology Transfer, Cen-Tre for Research, Rosenheim Technical University of Applied Sciences, Hochschulstraße 1, 83024 Rosenheim, Germany

**Keywords:** Critical Pathways, Implementation Science, Primary Health Care, Aged, Vertigo, Dizziness, General Practitioners, Physical Therapy Modalities

## Abstract

**Background:**

Vertigo, dizziness and balance disorders (VDB) are common in older people and cause restrictions in mobility and social participation. Due to a multifactorial aetiology, health care is often overutilised, but many patients are also treated insufficiently in primary care. The purpose of this study was to develop a care pathway as a complex intervention to improve mobility and participation in older people with VDB in primary care.

**Methods:**

The development process followed the *UK Medical Research Council guidance* using a mixed-methods design with individual and group interviews carried out with patients, physical therapists (PTs), general practitioners (GPs), nurses working in community care and a multi-professional expert panel to create a first draft of a care pathway (CPW) and implementation strategy using the *Consolidated Framework of Implementation Research* and the *Expert recommendations for Implementing Change*. Subsequently, small expert group modelling of specific components of the CPW was carried out, with GPs, medical specialists and PTs. The *Behaviour Change Wheel* was applied to design the intervention´s approach to behaviour change. To derive theoretical assumptions, we adopted *Kellogg´s Logic Model* to consolidate the hypothesized chain of causes leading to patient-relevant outcomes.

**Results:**

Individual interviews with patients showed that VDB symptoms need to be taken more seriously by GPs. Patients demanded age-specific treatment offers, group sessions or a continuous mentoring by a PT. GPs required a specific guideline for diagnostics and treatment options including psychosocial interventions. Specific assignment to and a standardized approach during physical therapy were desired by PTs. Nurses favoured a multi-professional documentation system. The structured three-day expert workshop resulted in a first draft of CPW and potential implementation strategies. Subsequent modelling resulted in a CPW with components and appropriate training materials for involved health professionals. A specific implementation strategy is now available.

**Conclusion:**

A mixed-methods design was suggested to be a suitable approach to develop a complex intervention and its implementation strategy. We will subsequently test the intervention for its acceptability and feasibility in a feasibility study accompanied by a comprehensive process evaluation to inform a subsequent effectiveness trial.

**Trial Registration:**

The research project is registered in “Projektdatenbank Versorgungsforschung Deutschland” (Project-ID: VfD_MobilE-PHY_17_003910; date of registration: 30.11.2017).

**Supplementary Information:**

The online version contains supplementary material available at 10.1186/s12875-021-01441-9.

## Background

Vertigo, dizziness and balance disorders (VDB) are frequent complaints of older people [1] and limit the capacity to pursue daily activities and social participation. The prevalence is reported with up to 50% in some trails [2, 3] and tends to increase with age [4]. Despite this, prevalence is difficult to describe exactly [5]. VDB represents one of the most relevant contributors to the burden of disability among older people living in the community in Germany and is associated with physical and psychological impact namely immobility, limitations in activities of daily living (ADL), decreased participation and lower psychological wellbeing [6–8]. Mobility restrictions of older people are distinct risk factors for falls [9] and even the fear of falling may lead to less activity and more disability [10]. Particularly in older people, the aetiology of VDB can rarely be attributed to distinct vestibular diseases, but more often to multifactorial deficits due to ageing, consequences of non-vestibular conditions or a combination of multiple aetiologies [3, 5, 11, 12]. VDB is a common reason for consulting a general practitioner (GP), affecting almost every person at least once in their lifetime [13]. A recent systematic review describes a prevalence of consultations for dizziness-related symptoms in primary care of approximately 1% to 15%, with benign paroxysmal positional vertigo (BPPV) being the most common specific aetiology in up to 40% [5]. In contrast to medical specialists, GPs see patients from the full range of medical disciplines and need to screen every patient to detect any serious health conditions. At the same time they treat many uncomplicated cases. Generally, GPs have to base their clinical reasoning process on the patient’s history and a few additional tests [5], whereas VDB are known to be described unclearly, inconsistently and unreliably by patients and are, thus, difficult to standardise [14]. To exclude life threatening health conditions, GPs have to use diagnostic procedures and referrals to specialists. On the other hand, overutilization of health care resources in patients with VDB insufficiently treated in primary care has been shown [15]. Most VDB cases can be improved by treatment [16] but often do not benefit from drug or surgical therapy [17]. Due to the multifactorial aetiology, this might be especially true for older patients. Physical therapy is a safe and effective treatment to promote mobility and avoid imbalance and falls [18]. Despite this, in the German guideline for acute VDB in primary care [19], physical therapy seems not to be a central treatment option, whereas a guideline for chronic VDB is lacking. Facing an ageing population due to demographic changes, strategies to manage VDB safely and efficiently are essential. Therefore, the management of VDB needs to be tailored for primary care with adequate referrals and treatment options [15].

A CPW is an evidence-based structured multidisciplinary care plan comprising all relevant diagnostic and therapeutic steps in the care of patients with a specific health condition in a chronological order [20]. CPWs are used to translate evidence into local practice by contemplating specific circumstances and demands; they tailor care and can reduce variations in practice to improve patient outcomes [20, 21]. CPWs for VDB should standardize GPs’ diagnostics, treatment options and referrals, and explicitly integrate the use of physical therapy prescriptions.

The aim of this study was to develop a CPW as a complex intervention to improve the mobility and social participation of older people with VDB in primary care by integrating existing evidence and stakeholders’ perspectives. Specifically, we aimed (a) to identify conditions of successful implementation by integrating the perspective of the involved health professionals regarding their expectations, attitudes, knowledge and needs, and (b) to address the issue of multi-professional communication by identification of supportive and hindering factors, and (c) to identify conditions of successful implementation by integrating the consumers’ perspective regarding their experiences about accessibility and availability, expectations, motivation and beliefs.

## Methods and results

**Fig. 1:**
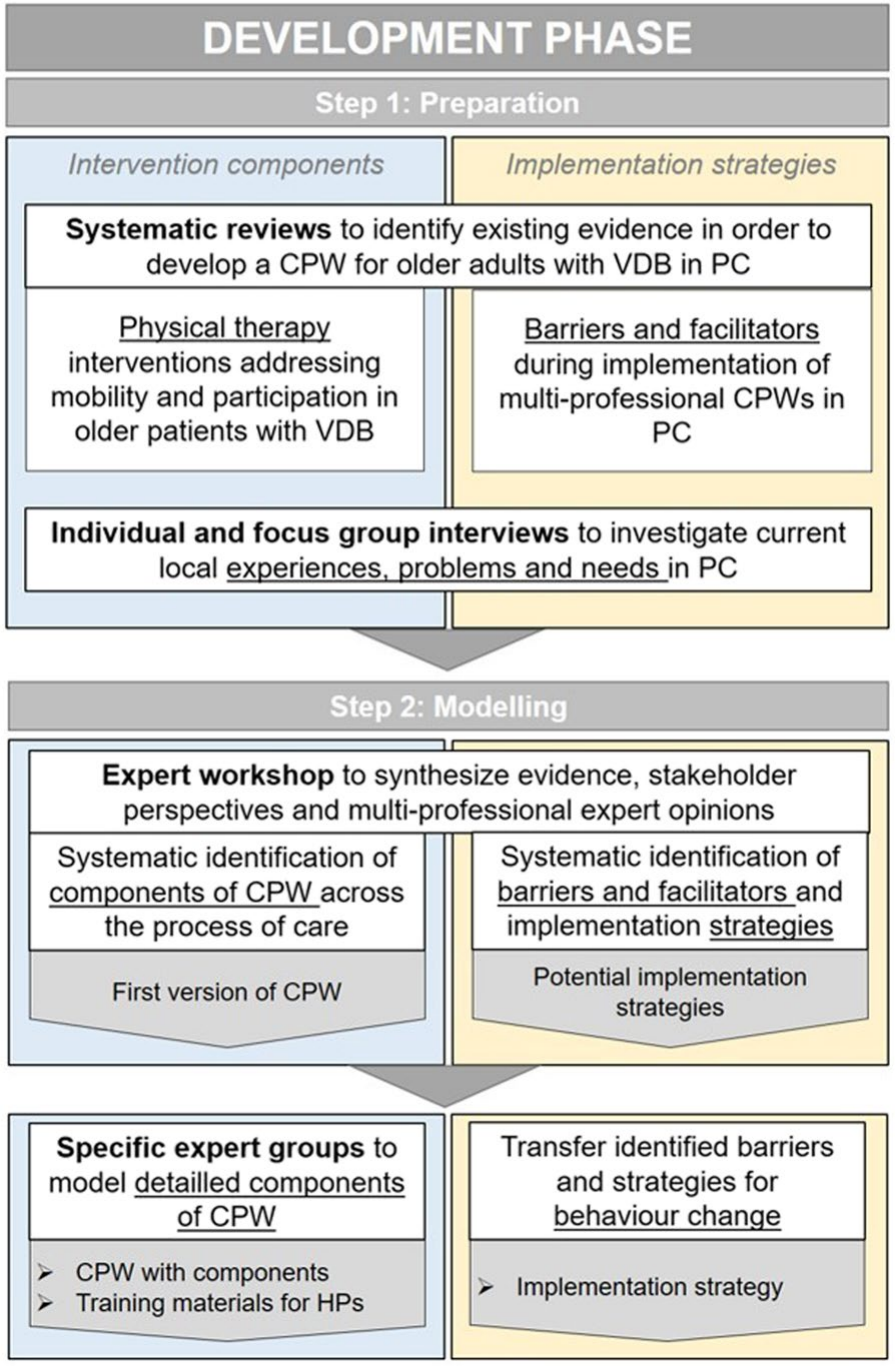
Aims and methods for development of intervention components and implementation strategies of intervention. Legend: CPW = Care pathway; HPs = Health professionals; PC = Primary care; VDB = Vertigo, dizziness and balance disorders

We developed our CPW as a complex intervention according to the *UK Medical Research Council (MRC) framework* [22], which provides a methodological framework to develop, pilot test, evaluate and implement complex interventions. This paper describes the first phase of this framework, the development phase. It was carried out in a stepwise process divided into two sub-phases: First, a preparation phase that included the identification of existing evidence and of stakeholders’ and consumers’ demands was conducted followed by a modelling phase, that comprised the modelling of a CPW and an implementation strategy. The feasibility and piloting study will be reported elsewhere. An overview of the specific aims and methods of the development steps is shown in Fig. 1.

To report the development of the complex intervention we used the *Criteria for Reporting the Development and Evaluation of Complex Interventions in healthcare* [23] (s. Additional file 1).

## Step 1: Preparation

### Methods step 1

#### Identifying existing evidence and theory

The first step included a synthesis of existing evidence by carrying out two systematic reviews to identify existing evidence. First, a systematic review to identify the quality of evidence of physical therapy interventions addressing mobility and participation in older patients with VDB was conducted [24]. Due to the heterogeneity of the included studies, a narrative synthesis across all types of interventions was conducted concerning the outcome measures covering aspects of the *International Classification of Functioning, Disability and Health (ICF)* [25], quality of life and general health. Second, barriers to and facilitators of successful implementation of multi-professional CPWs in primary care were investigated [26]. Due to the large diversity of study characteristics, interventions and outcomes, we carried out a narrative synthesis following the *guidance for undertaking reviews in health care* from the *Centre for Reviews and Dissemination* [27].

#### Exploring the health care providers´ perspective

To develop a tailored and successful CPW we used the co-creation approach by considering the perspectives of all involved groups, collaborating with them to allow the inclusion of their different needs and perspectives and to support development regarding designs and content [28]. Health care providers were selected by identifying health professionals involved in the process of care of patients with VDB. It was assumed that GP practices were the first place to go for people aged at least 65 years with beginning VDB problems or, in case of home care, community nursing services, who seem to act as gatekeepers for the following patient care process. PTs might offer interventions for patients with VDB. The target population was defined as patients older than 65 years suffering from VDB.

#### Individual interviews with health professionals

To explore current practice and identify needs for improvement, the ideal patient trajectory, expectations, attitudes and knowledge, we carried out semi- structured individual interviews with health professionals involved in the primary care of VDB patients: GPs, PTs and nurses working in a community setting.

#### Group interviews with health professionals

To address the issue of multi-professional communication and to identify supportive and hindering factors, we conducted interprofessional focus group interviews among health professionals involved in the primary care of VDB patients: GPs, PTs and nurses.

#### Study design, recruitment, data collection and analysis

We aimed to recruit approximately 10 GPs, 10 PTs and 10 nurses for the individual interviews. When data saturation from individual interviews was reached, we asked the remaining participants to participate in a focus group interview. We initially planned 2 focus groups with a balanced number of each health care professional group: 1 GP, PT and nurse each. For both individual and focus group interviews, we included health professionals with at least three years of clinical experience in primary care, outpatient practices, or community/home care services. Further inclusion criteria were self-rated specific and extended experience in the treatment and management of older patients experiencing VDB and written consent to participate in the study. We searched for potential health professionals via the internet and regional networks, used telephone requests and, for those interested, sent further information via email or fax, as preferred.

Participants gave their written consent for participation prior to the date of the interview. The interviewers had a clear structured guideline for every kind of interview and interviewee, with defined main questions and examples for requests. The interviewers tried to systematically moderate the interviews and maximize inter-interviewer reliability. The interview guide for patients and GPs is provided as an example of the guide for health professionals (see Additional file 2). All interviews were audio-recorded, and field notes were taken during all interviews. Afterwards, the audio records were transcribed verbatim according to the rules of Kuckartz [29] with transcription software F4 (https://www.audiotranskription.de/f4). Data was analysed using structuring qualitative content analysis [29] to identify common themes on issues and barriers and facilitators to multi-professional communication. Two researchers (VR and ES) independently carried out a first draft of a coding tree using MAXQDA software (https://www.maxqda.de/) for every kind of interview setting and participant group and then discussed differences. Subsequently, VR and ES coded the material and included field notes in the analysis. Data saturation was defined as the point when no additional information was obtained.

### Exploring the health care consumers´ perspective

#### Individual interviews with patients

To identify conditions of successful CPW implementation by integrating the consumers’ perspective (experiences regarding accessibility and availability, expectations, motivation and beliefs) into the development process we conducted individual interviews with affected people.

#### Study design, recruitment, data collection and analysis

We included patients aged at least 65 years who consulted a GP with complaints of VDB. Additionally, we recruited patients in PT practices, who are already enrolled in PT programmes. Patients were approached by putting up a poster in PT practices to recruit affected people and providing our telephone number for further information in case of interest. Patients became aware of the opportunity to participate because of their PT or relatives/ an acquaintance. Exclusion criteria were patients aged under 65 years, patients having a serious condition/disease, patients requiring hospital treatment, and patients wishing to be excluded from the study. Additionally, we investigated the impact of VDB symptoms on the patients’ activities of daily living and social participation using the German version of the vertigo activities and participation questionnaire (VAP) [30].

Participants gave their written consent for participation prior to the date of interview. The interviewers had a clearly structured guideline with defined main questions and examples for requests and, thus tried to systematically moderate the interviews and minimize inter-interviewer reliability. Interviews were audio-recorded and field notes were taken during all interviews. Afterwards, the audio records were transcribed verbatim according to the rules of Kuckartz [26] with transcription software F4 (https://www.audiotranskription.de/f4). Data was analysed using structuring qualitative content analysis [29] to identify common themes on issues and barriers and facilitators to multi-professional communication. Two researchers (VR, ES) independently carried out a first draft of a coding tree using MAXQDA software (https://www.maxqda.de/) and then discussed the differences. Subsequently, VR and ES coded the material and integrated their field notes into the analysis according to Kuckartz [29]. There, we aimed to gain knowledge about the respective issues and reported results narratively. Data saturation was defined as the point, at which no additional information was obtained. For the VAP questionnaire we calculated descriptive statistics.

#### Characteristics of the interviewers

The two researchers (ES and VR, both master’s degree and vocational training as health professionals) conducted the interviews and the study assistant wrote the protocols. Both interviewers were trained how to develop an interview guideline and perform interviews by a separate qualitative workshop and had further experience from prior research activities. No relationships were established with participants prior to study commencement. All participants were informed about data privacy and about the intentions of doing this research prior to the interview.

## Results step 1

### Results of systematic reviews

The systematic review revealed that for older people, active physical therapy using vestibular rehabilitation, regardless of any variations and in combination with repositioning manoeuvres was most effective [24]. The second review identified barriers to and facilitators of successful implementation of CPWs within the context, implementation and setting dimensions of *Context and Implementation of Complex Interventions (CICI) framework* dimensions [31], which need to be considered in the implementation of CPWs [26]. Detailed results of systematic reviews are published elsewhere [24, 26].

### Results of the individual and focus group interviews with health professionals

#### Characteristics of the interviewees

Of the 35 invited GPs, 9 consented to participate (response rate: 26%); 7 participated in individual interviews and 2 participated in the focus group interviews. Of the 14 PTs invited, 8 consented to participate (response rate: 57%). Among those, 6 participated in individual interviews and 2 participated in focus group interviews. A total of 9 nurses were invited, and 7 participated (response rate: 77%): 5 in individual interviews and 2 in focus group interviews. Reasons for non-participation were holidays, lack of time or staff shortage.

After the analysis of a total of 17 health professionals (7 GPs, 6 PTs, 5 nurses) individual interviews, no further aspects or themes emerged and data saturation was reached. Most individual interviews were conducted via telephone to participants being in their institution or at home (n = 16, 94%), and only one PT preferred a face-to-face interview at the study centre. Interviews lasted 11 to 29 min. The characteristics of the health professionals are shown in Table 1.

**Table 1:** Characteristics of health professionals who participated in individual interviews

	**GPs** (*n* = 7)	**PTs** (*n* = 6)	**Nurses** (*n* = 5)
Age (Mean ± SD (Range))	58.7 ± 7.87 (42 – 66)	42.0 ± 10.71 (28 – 58)	43.2 ± 11.05 (31 – 55)
Sex (female / %)	3 / 43%	5 / 71%	3 / 60%
Years of current occupation (Mean ± SD (Range))	30.0 ± 8.04 (14 – 37)	18.3 ± 10.39 (6—36)	20 ± 5.87 (15 – 30)
As community nurse (Mean ± SD (Range))	n.a	n.a	12.8 ± 10.85 (1 – 27)
Weekly hours with patients (Mean ± SD (Range))	n.a	34.8 ± 8.21 (6 – 36)	30 ± 21.11 (1 – 60)

*GPs* General practitioners, *n.a.* not assessed, *PTs* Physical therapists, *SD* Standard deviation

After the analysis of the two focus group interviews with one GP, PT and nurse each no further aspects or themes emerged and data saturation was reached. Both group interviews were carried out for a duration of 53 min each. The focus group interviews were conducted face-to-face. The characteristics of the health professionals are shown in Table 2.

**Table 2:** Characteristics of the focus group interview participants

	**FG 1**					**FG 2**				
	**GP** (*n* = 1)		**PT** (*n* = 1)	**Nurse** (*n* = 1)		**GP** (*n* = 1)	**PT** (*n* = 1)		**Nurse** (*n* = 1)	
Age (years)	68	50			55	66		27		31
Sex (m / f)	m	m			f	m		f		f
Years of current occupation (years)	40	25			25	41		8		7
As CN (years)	n.a	n.a			n.s	n.a		n.a		4
Working hours per week (%)	110	100			70	130		100		100

*CN* Community nurse, *f* Female, *FG* Focus group, *GP* General practitioner, *m* Male, *n.a.* not assessed, *n.s.* not stated, *PT* Physical therapist

#### GP perspective

GPs see their role as gatekeepers and complain that it is difficult to act in this role after the patient has been referred to a specialist. They described an ideal patient trajectory as efficient, fast and comprising a diagnostic work up in a multidisciplinary center. Some GPs recommended, the CPW should involve a broader treatment approach including psychological coaching and social interventions, such as a pensioners´ exchange or multipurpose associations published in a GP practice.

#### PT perspective

From the PT perspective, the most relevant problem was that referrals from GPs are mostly without proper information on the physicians’ diagnostic results. Therefore, PTs have to identify the patients’ problems without this information. However, a specific German indication key (SO3—physical therapy with indication for dizziness of different origins and aetiology) was not used by the GPs.

*“I think they* (the GPs) *are hardly informed about what they can assign to what kind of patients (…) I can remember only one patient (…) coming with this (…) indication key (…). All other* (patients are assigned concerning) *cervical spine.”* (PT, interviewee 4).

PTs reported mixed confidence in their abilities to treat VDB patients. They identified their knowledge from vocational training as less relevant and relied on skills acquired by additional trainings instead. Interdisciplinary communication, especially with GPs, was rated as insufficient and the PTs assumed that therapy reports were hardly read by GPs. A structured approach tailored to the needs of VDB patients was considered to be helpful:

*“For certain things sometimes there exists very clear and beautiful guidelines, like a catalogue where you choose (…) I have a tree (…) something like a decision tree, exactly.”* (PT, interviewee 2)

PTs rated more specific educational training, interdisciplinary cooperation and patient information to be beneficial.

#### Nurses´ perspective

From the perspective of community nurses, a main problem is finding PTs that are available for home visits. It was also criticized, that interdisciplinary communication is hampered by missing reimbursement or financial incentives. The ideal would be an interdisciplinary documentation system.

*“They should document or record everything. Either that or have a kind of online portal; that would of course be the easiest. That means, where you can exchange information about the patient and (.) where everyone can write something there or in the documentation folders on site. In the end, it takes just a minute that you write in there.”* (Nurse, interviewee 14).

In addition, specific educational training for nurses, and programmes to promote faster support for affected people was identified to be helpful.

#### Multidisciplinary perspective

It was mentioned in both focus groups that an important barrier for good multiprofessional and patient-centred communication is that there is no additional reimbursement for such activities. It was mentioned as critical that GPs do not have a central gatekeeper role when the first contact point of a patient was a medical specialist. Knowing each other personally was identified as the most relevant facilitator of good cooperation, e. g when GPs or PTs are organised in centres. Space for potential improvement is seen in the communication between GPs and PTs, in particular, interdisciplinary case conferences with video conferencing and digital shared online documentation were seen as potentially helpful.

*“I think the online portal is the one thing that could best be realized. (…) time is relatively tight (…) and you do not have to sit down together, you can actually do it online. In addition, maybe just write to me. So I find that feasible now.”* (Nurse, interviewee 1)

*“So, I think team meetings are less feasible because the different times can never be brought together (…) it is of course also unpaid time. (…) the basis could be an electronic document (…) and 80% can then be resolved (…) and the rest, you are (…) on the phone.* (GP, interviewee 1)

In summary, the optimal health care strategy for older VDB patients was described as long-term, continuous and target-group-specific.

### Results of the individual interviews with patients

#### Characteristics of the interviewees

A total of 14 patients contacted the study centre because of interest and 11 consented to participate (patients’ response rate: 79%). Reasons for non-participation were lack of interest or time.

At the point of data saturation, a total of 11 patients participated in individual interviews, which were conducted via telephone (n = 10; 91%); only one patient preferred a face-to-face interview at the study centre. In some cases, the patients’ partners joined the telephone interview if the interviewee had impaired hearing abilities. The interviews lasted 13 to 33 min. Characteristics of participants are shown in Table 3.

**Table 3:** Characteristics of patients participating in individual interviews

	**Patients** (*n* = 11)
Age (Mean ± SD (Range))	75.5 ± 6.9 (65 – 89)
Sex (female / %)	7 / 64%
Symptoms (n / %):	
Dizziness	9 / 82%
Balance disorder	4 / 36%
Gait instability	10 / 91%
Fall history	8 / 73%
Other additional symptoms	6 / 55%

*SD* Standard deviation

#### Patient perspective

Some patients reported not having consulted any physician because they considered their symptoms as not very serious or lacked time for a visit. From the patients’ perspective, optimal health care was described by all interviewed patients when symptoms are taken seriously by GPs and are not only attributed to ageing. Therapy should include continuous PT, home training, conventional and alternative therapy approaches, medication, age specific offers or group sessions.

## Step 2: Modelling

### Methods step 2

#### Modelling the first version of the CPW and implementation strategies

##### Expert workshop

The expert workshop was planned as a three-day event in a closed setting to create a productive and focused working space. An external moderator was introduced to the subject and process of the expert workshop.

##### Study design, recruitment, data collection and analysis

We aimed to recruit all experts and at least one of them who are involved in the process of care of our target group. Therefore, we identified potentially involved persons following the *International Classification of Functioning, Disability and Health (ICF)* rehab-cycle [32] as a basic theory of structuring the patient´s rehabilitation process and characterizing the steps of involved health professionals. In addition to the participants in the interviews, we identified health professionals such as clinical experts, representatives of health insurances, of health care researchers and of affected individuals.

To recruit participants, collaboration partners and local practices/institutes were contacted. Recruitment of health insurance representatives was conducted via personal contacts. We re-recruited interview partners and used member lists of the *Association of Statutory Health Insurance Physicians*. Self-employed persons received remuneration for the time spent participating in the workshop.

##### Methods of the workshop

**Fig. 2:**
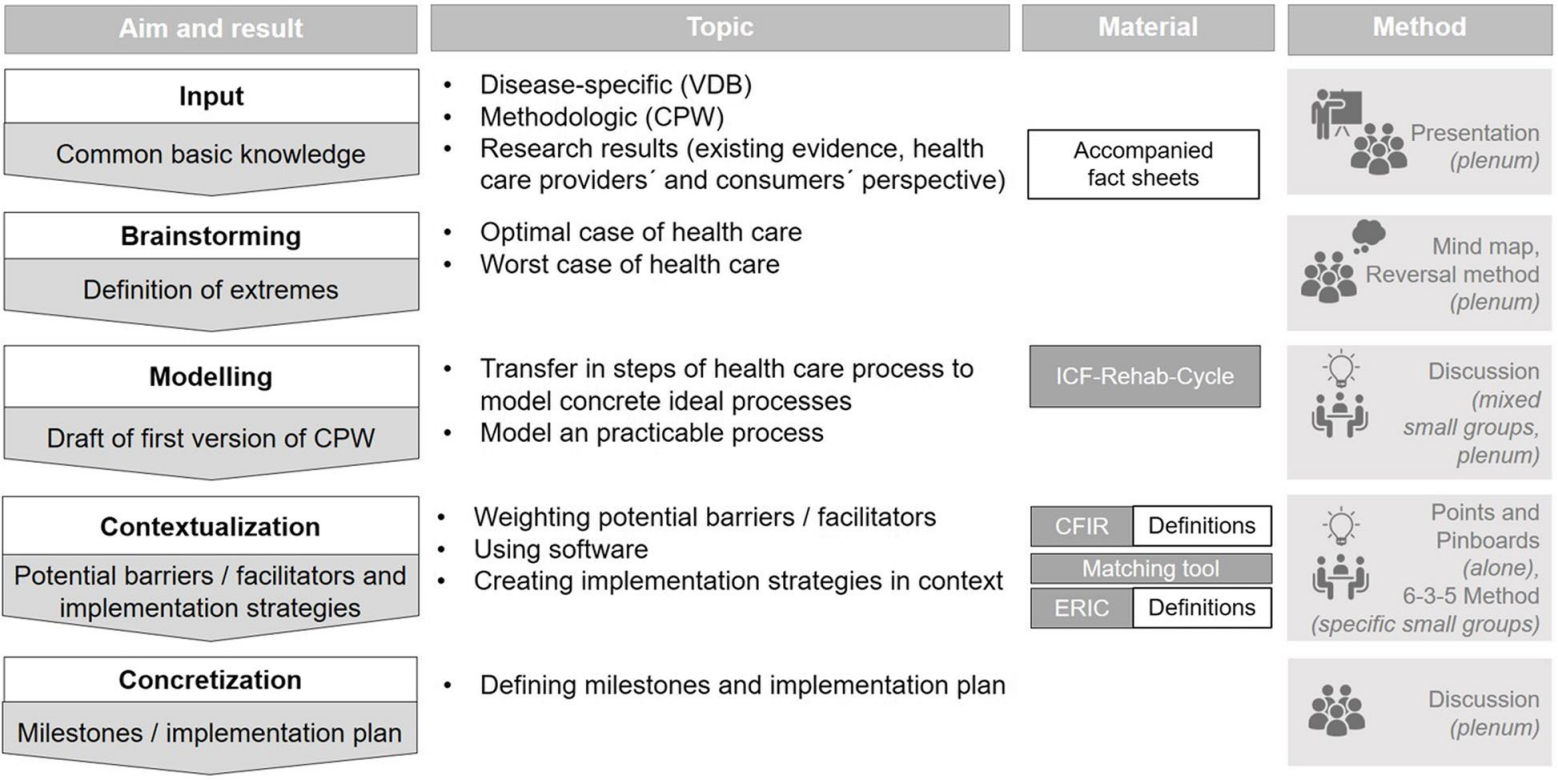
Process of the expert workshop. Legend: CFIR = Consolidated framework of implementation research; CPW = Care pathway; ERIC = Expert recommendations for implementing change; ICF = International classification of functioning, disability and health; VDB = Vertigo, dizziness and balance disorders. “Specific groups” means 1 kind of experts in 1 group; “Mixed groups” means every kind of experts in 1 group

At the expert workshop, a stepwise modelling process was conducted (see Fig. 2). First, an update on recent disease-specific knowledge was given by a senior medical doctor and methodologic introduction to CPWs was given by a health care researcher. This was necessary to start with a common basic knowledge base among the participating experts. Then, the results of prior research were presented by the research team and accompanied by factsheets: The results of existing evidence (systematic reviews) and of health care providers´ and consumers´ perspectives (interviews). To guide experts through different stages of the modelling process various creative techniques in the plenary session, individually and in small groups, were used. Good evidence exists for the *Consolidated Framework of Implementation Research (CFIR)* [33] and *Expert Recommendations for Implementing Change (ERIC)* [34] as well as for a matching tool to both systematically identify potential barriers/facilitators and select implementation strategies for interventions. To identify potential barriers of and facilitators to by implementing a CPW in a real-world setting, CFIR barriers were used and prioritized by the experts. These barriers were transformed by the CFIR/ERIC matching tool into a weighted order of matching ERIC strategies. The frameworks were translated into the German language and the translation will be published elsewhere. In conclusion, milestones and an implementation plan were defined.

### Subsequent modelling of the CPW, intervention components and educational training materials

#### Modelling process of CPW design in a specific expert group

The results of the expert workshop were collected, reviewed and analysed. According to defined milestones, a checklist for GPs and a guide for PTs with accompanied educational trainings were developed. In two expert meetings, we modelled a more detailed version of the GP’s role in the CPW in an iterative way. Subsequent to each of the two face-to-face meetings, feedback and further exchanges via telephone or email with the research team was done if necessary. Based on the ICF-Rehab cycle and its process of care as well as the literature regarding evidence-based practice [35], the research team developed a first draft of an algorithm for the PT-guide. In telephone contacts with renowned PT specialists an enhanced version was adopted.

#### Modelling behaviour change

We conducted a stepwise approach to intervention design and implementation strategy using the *Behaviour Change Wheel (BCW)* [36] to guide the approach. We also took barriers and facilitators identified according to CFIR [33] and matched implementation strategies according to ERIC [34] from our expert panel into consideration. In an iterative way, we moved between the BCW and CFIR/ERIC. These frameworks helped to organize and develop specific behaviour change techniques and implementation strategies. To design behaviour change, we conducted the 7 steps of the guide using the provided worksheets (see Additional file 3). We applied the *Capability-Opportunity-Motivation-Behaviour (COM-B)* model and *Theoretical Domains Framework* and used potential barriers according to the CFIR and ERIC strategies from our expert workshop.

#### Developing a Logic Model

To systematically present the relationships between the intended results, the underlying mechanism of change and the planned work, we developed a logic model according to *Kellogg´s Logic Model Development Guide* [37]. Finally, we checked the model for its completeness regarding context, implementation and setting with the *Context and Implementation of Complex Interventions Framework* [31].

## Results Step 2

### Results of the expert workshop

#### Characteristics of the experts

The expert workshop was conducted for three days in October 2018. The response rates of clinical experts, researchers and representatives of affected people and insurances were 50% to 100%. We had problems recruiting GPs (response rate: 0.1%): When recruiting regional GPs, the response rate was 14% (1 participating GP out of 7 requests), but the response when re-recruiting interview partners (9 requests) and using member lists of the *Association of Statutory Health Insurance Physicians* (124 requests) was 0%. In total, 9 clinical experts (2 neurologists, 1 ENT physician, 1 GP, 3 PTs, 1 geriatric nurse, 1 medical assistant), 2 experts in health care research, 2 health insurance representatives, and 2 patient representatives participated.

#### First version of the CPW

Experts drafted a first version of the CPW according to the steps of the ICF Rehab cycle [32]. Regarding access, a hotline for patients and population-related information was recommended. This version included tools for health professionals to screen, assign, treat and evaluate VDB patients and was intended to promote multi-disciplinary communication between all involved HPs.

### Implementation strategy

#### Potential barriers and facilitators

**Fig. 3:**
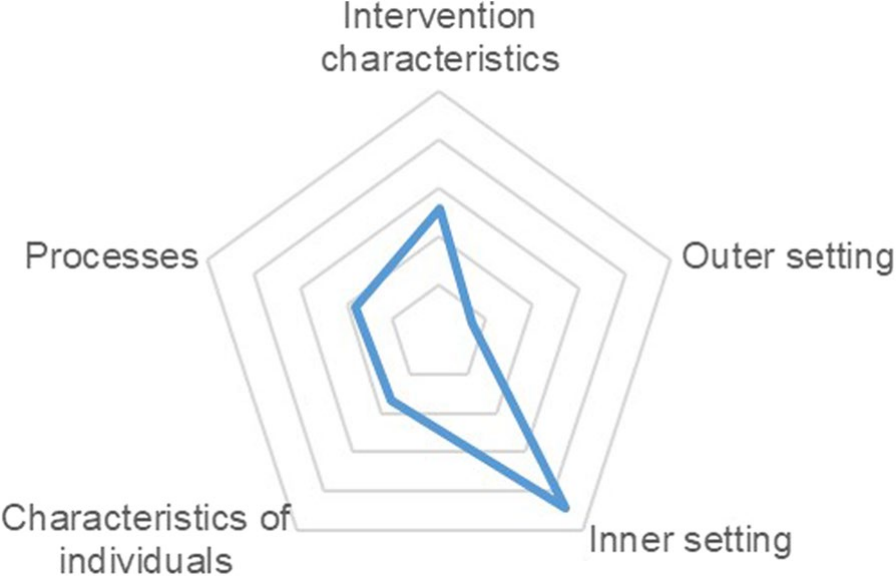
Identified barriers by the expert panel

Weighting potential barriers and facilitators according to the CFIR [33] the most prioritized construct was the inner setting (44 points; 39%), followed by intervention characteristics (26 points; 23%), processes (18 points; 16%), characteristics of individuals (17 points; 15%) and outer setting (7 points; 6%) (see Fig. 3, details see Table 4).

**Table 4:** Potential barriers and matched implementation strategies

CFIR			ERIC	
**Priority**	**Construct**	**Barrier**	**Priority**	**Strategy**
1	Intervention characteristics	Cost	1	Identify and prepare champions
2	Inner setting	Organizational Incentives & Rewards	2	Alter incentive/allowance structures
3	Characteristics of individuals	Knowledge & Beliefs about the Intervention	3	Assess for readiness and identify barriers and facilitators
4	Processes	Reflecting and evaluating	4	Conduct local consensus discussions
5	Inner setting	Implementation climate	5	Inform local opinion leaders
6	Inner setting	Available resources	6	Conduct educational meetings
7	Processes	Planning	7	Access new funding
8	Intervention characteristics	Evidence strength & quality	8	Capture and share local knowledge
9	Outer setting	External policy & incentives	9	Conduct local needs assessment
10	Characteristics of individuals	Individual stage of change	10	Develop a formal implementation blueprint
11	Intervention characteristics	Relative advantage	11	Audit and provide feedback
12	Inner setting	Tension for Change	12	Build a coalition
13	Inner setting	Goals and Feedback	13	Develop and implement tools for quality monitoring
14	Inner setting	Leadership Engagement	14	Identify early adopters
			15	Involve executive boards

*CFIR* Consolidated framework for implementation research, *ERIC* Expert recommendation for implementing change

### Implementation strategies

After transformation of these barriers using the matching tool software, a weighted order of matching ERIC strategies was presented and the 15 most important strategies were further elaborated (see Table 4). From them, the experts could choose, specify and discuss which implementation strategy would be appropriate to implement the CPW. It was also possible to deselect a strategy if the strategy did not match the implementation context.

### Milestones and implementation plan

The implementation plan with milestones for the subsequent study was discussed and approved by the experts. Regarding access, recruitment of potential study participants should be conducted by GPs using clear inclusion criteria for patients. Assessment and assignment should be conducted by GPs using a screening tool with accompanied and credited educational training. Regarding assignment and evaluation, no milestones were defined. To evaluate the effectiveness of the (assigned) intervention, specific follow-up times were recommended. For detail see Table 5.

**Table 5:** Steps of the CPW and milestones of its implementation

Steps of the CPW	Milestones
Access:	
- Immediately	→ Reaching of the pre-defined number of participating GPs and patients
- Direct	
- Involvement of relatives	
Assessment:	
- Central role of GP	→ Development of a screening tool
- Checklist	→ Accompanied previous educational training of GPs
- Educational training of GPs	
Assignment:	
- Prompt	
- Physical therapy as favoured intervention	
- Organized in a network	
- Case managers	
Intervention:	
- Capacity of providers	→ Recruitment of collaboration partners
- Treatment duration	→ Educational training of PTs
- Involvement of additional potential actors	
- Extended offer of providers (e.g., clubs, community college)	
Evaluation:	
- Feedback to all involved actors	
- Follow-up appointments in checklist	

*CPW* Care pathway, *GPs* General practitioners, *PTs* Physical therapists

### Results of subsequent modelling design and implementation of the CPW

#### Logic Model of the CPW´s intervention components and implementation strategies

**Fig. 4:**
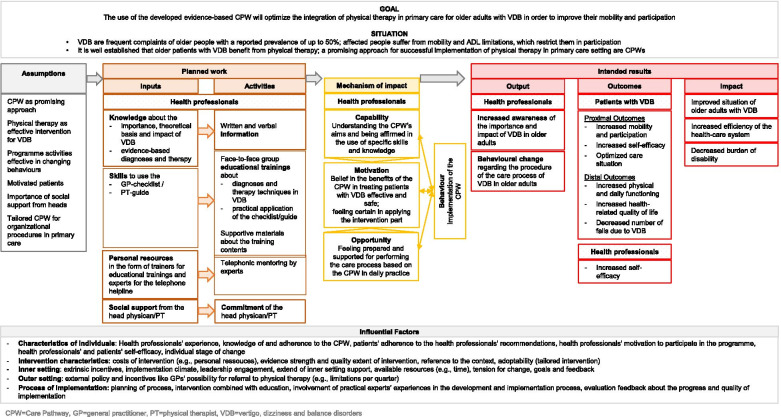
Logic Model underlying the CPW. Legend: ADL = Activities of daily living; CPW = Care pathway; GP = General practitioner; PT = Physical therapist; VDB = Vertigo, dizziness and balance disorders

We developed a logic model by using Kellogg´s Logic Model (see Fig. 4) and combining the findings of prior results regarding assumptions and influential factors. Since the key to practice development is behaviour change among health professionals, we defined the planned work, mechanism of impact (using the COM-B model, the inner layer of the BCW [28]) and intended results. To improve VDB patients´ mobility and participation, we aim to promote the self-efficacy of health professionals by supporting them in behaviour change to use the CPW. Therefore, we plan to give them more in-depth knowledge and skills via written information and face-to-face educational trainings in how to diagnose and treat VDB patients efficiently using distinct parts of the CPW (GP-checklist and PT-guide). The mechanisms of impact can be explicated as follows: When the health professionals understand the aims of the CPW and are affirmed in the use of specific skills and knowledge (capability), if they feel prepared and supported for performing the care process based on the CPW in daily practice (opportunity), they can believe in the benefits of the CPW in treating patients with VDB effective and safe and can feel certain in applying the intervention part (motivation) and then will implement the CPW.

#### Characteristics of the small expert group

The small expert group consisted of the participants of the expert workshop: 1 GP, 1 neurologist, 1 neuro-otologist and 1 ENT physician and the research team. For the telephone contacts, 1 PT from the expert workshop and 2 additional renowned PT specialists from collaborating partners participated.

#### CPW

**Fig. 5:**
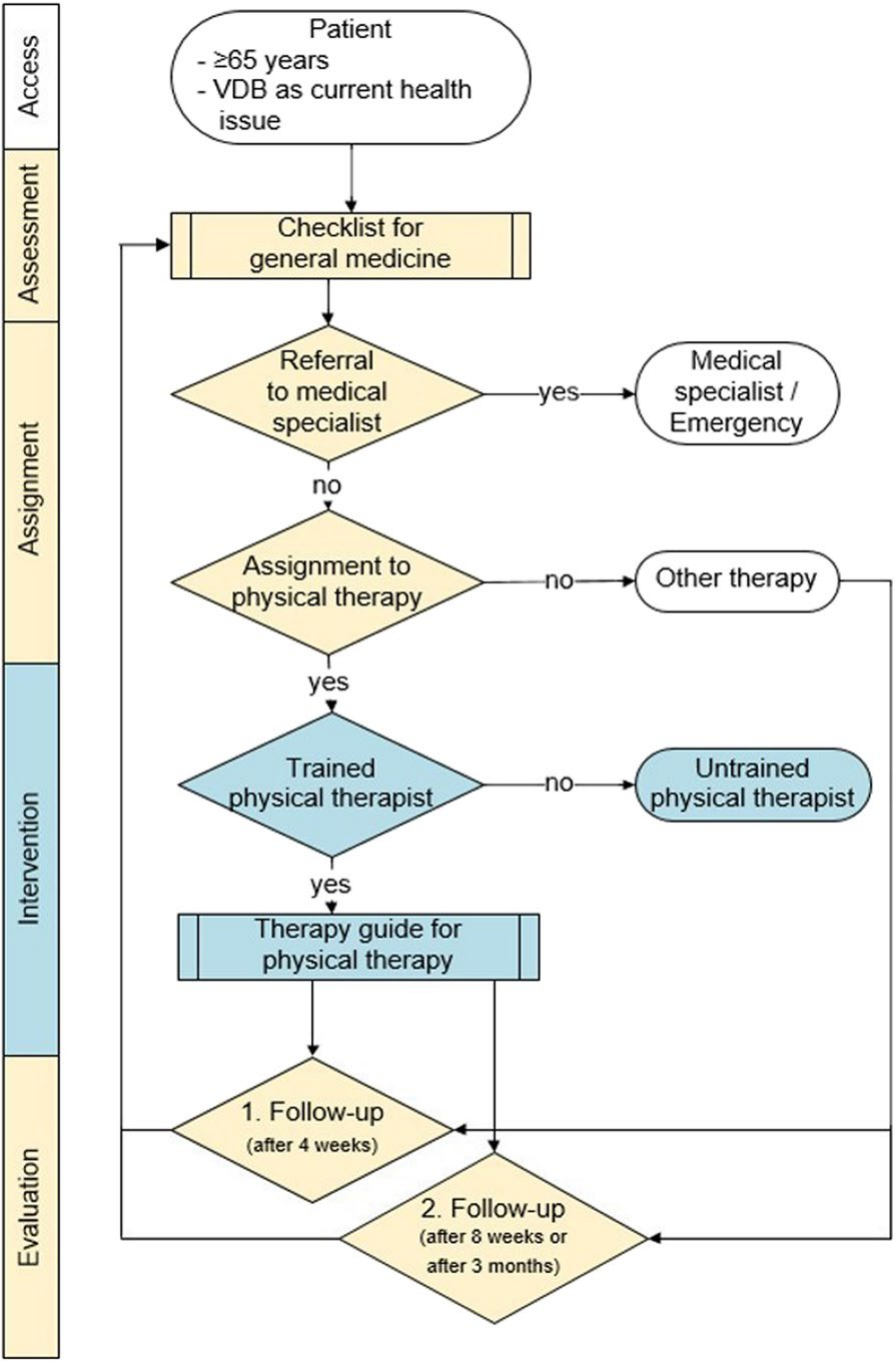
Multi-disciplinary CPW for older people with VDB in primary care*.* Legend:  = Task of general practitioner;  = Task of physical therapist;  = Start;  = Decision;  = Sub-process

The developed multi-disciplinary CPW is a paper-based algorithm, that illustrates all steps of the aged patients’ care path in a structured way (see Fig. 5). The specific subprocesses of the CPW are a checklist for GPs and a guide for PTs which are described in detail in the following section:

### Intervention components and educational training materials

#### GP checklist

The screening checklist for GPs that manage patients with VDB aims (a) to exclude life-threatening conditions (b) to promote reliable diagnosis and evidence-based treatment by GPs and (c) to ensure a rational referral regime. The final checklist is a paper-based algorithm and consists of aspects of anamnesis, assessments, specific referral regimes (e.g. prescription guide for physical therapy) and assignments to therapy and follow-up timelines for consultation. The checklist is not available since it has not yet been evaluated for effectiveness and safety. Linking to our Logic Model, we use the checklist as an input on GPs´ knowledge and skills to change their behavior in respect to having an increased awareness of the importance of VDB in older people and their procedure of care (output). As an activity to reach this behavior change, we provide an educational training for GPs. This training aims to develop an in-depth understanding of the checklist and exercises.

#### PT guide

An evidence-based guide for clinical reasoning and treatment for PTs focusing on the leading symptoms of chronic dizziness and balance disorders. The guide is not available since it has not yet been evaluated for effectiveness and safety. The guide contains guidance on the prescription header (specific code and assignment of the GP), anamnesis, assessment, treatment and evaluation. Regarding anamnesis, background information about clinical patterns was included. The decision tree style leads to specific assessments and treatment options. Additionally, educational flyers and leaflets were included to be handed out to patients during the therapy: 4 educational leaflets about practical exercises (physical therapy for balance disorders, gait disorder and vertigo as well as instructions for the positioning manoeuvre of posterior canal benign paroxysmal positional vertigo) provided through collaboration with the *German Centre for Vertigo and Balance Disorders* and 2 informational flyers that were translated from existing literature into the German language. These flyers include topics such as symptom control of vertigo and nausea [38] and frequently asked questions about benign paroxysmal positional vertigo [39] using the American clinical practice guideline [40].

As was done for the GP checklist, we used the PT guide as an input on PTs´ knowledge and skills to change their behavior. PTs should be aware of the importance of VDB in older people and their approach to physical therapy (output). To ensure correct and confident application of the guide including assessments and interventions, educational training was performed as an activity. Prior to the training, informational material, was provided to prepare the participants and ensure a common base of knowledge.

#### Implementation strategy of the CPW

As a result, behaviour change of health professionals is needed to apply evidence-based checklists or guides. The implementation strategy comprises an face-to-face educational training in groups, accompanying information or instruction manuals and social support by mentoring during the first phase of application and by providing a phone helpline at an individual-level. A material incentive such as accreditation points for educational training or case payments seemed to be useful for participation.

## Discussion

### Main findings

We developed a theoretically-based and practically-informed CPW as a complex intervention to improve the mobility and social participation of older people with VDB in primary care, which can now be tested for its feasibility. This intervention is based on findings from the literature, the perspective of older people with VDB and the experiences of health professionals working in primary care with these patients. In an expert workshop and subsequent small expert workshops, a CPW and its components were modelled, and specific implementation strategies were defined: A checklist for GPs and a guide for PTs working in primary care with accompanied educational trainings to support the use of an evidence-based standardized approach to VDB patients in daily practice.

The optimized integration of physical therapy in the primary care of VDB patients and a subsequent, targeted promotion of physical activity of affected people is a central aspect of our intervention. Findings from the literature show promising effects of complex interventions in supporting older people to live independently in the community, reducing nursing-home and hospital admissions and the occurrence of falls, increasing physical function and health-related quality of life, especially, because these interventions can be tailored to meet individuals' needs [41, 42].

By collecting data using a mixed-methods design, we were able to identify not only complementary evidence, but also to integrate different perspectives of all stakeholders. Rousseau et al. [43] highlighted the importance of paying attention to study design when developing a complex intervention and capturing different types of knowledge during the design progress to maximise creativity. We also confirm the mixed-methods design as a feasible developmental design to gather insights and understanding [44].

For the development of the CPW components, we used an approach, where we involved stakeholders, which is known to have high potential for societal impact via community-academic partnerships [45]. Involving stakeholders and undertaking primary data collection is also crucial in creating an acceptable and real-world intervention. We involved both those who are targeted by the intervention (patients) and those who are involved in its delivery (health professionals), which is a clear strength of this study.

The complex intervention was developed according to the *UK MRC Framework* [22], which explicitly gives reason about what and how the intervention should be implemented, additionally, Kellogg´s logic model [37] helped us to understand how the intervention might work and what activities are needed.

### Strengths and limitations

A clear strength of our study is that we conducted development based on a broad range of sources: The review of published research evidence, primary data collection with different stakeholders to explore their needs and understand context, and involvement of stakeholders in the iterative modelling process resulting in a logic model. These activities are in line with a recently published development guidance [46].

The participation of GPs in the expert workshop was hard to realize. Extensive and time-consuming recruitment resulted in only one participating GP. As we used the co-creation approach, the opinion of GPs who subsequently apply the CPW might be biased and misinterpreted due to the participation of too few GPs in our development process. To evaluate the GPs’ contribution to the developed intervention and its implementation strategy we firmly plan to include the participating GPs of the feasibility study into the process evaluation in a combined qualitative group interview together with the developers and to further incorporate their feedback.

## Conclusion

The complex intervention to improve mobility and participation of older people with vertigo, dizziness and balance disorders in primary care is now ready for feasibility testing. This step should be used prior to the main trial for assessment of its effectiveness and accompanied by a comprehensive process evaluation to identify experiences, relevant influences and explore barriers to and facilitators of successful implementation.

## Supplementary Information

Additional file 1

Additional file 2

Additional file 3

## Data Availability

All data generated or analysed and the measurements used during this study, not included in this report, are available from the authors on request.

## Abbreviations

BCW: Behaviour change wheel
CFIR: Consolidated framework for implementation research
COM-B: Capability-Opportunity-Motivation-Behaviour
CPW: Care pathway
ENT: Ear-nose-throat
ERIC: Expert recommendation for implementing change
GP: General practitioner
ICF: International classification of functioning, disability and health
P: Patient
PT: Physical therapist
VDB: Vertigo, dizziness and balance disorders
