# Supplementary material for: Development of a complex intervention to improve mobility and participation of older people with vertigo, dizziness and balance disorders in primary care: a mixed methods study
Source: BMC Fam Pract. 2021 May 12;22:89. doi: 10.1186/s12875-021-01441-9 (PMC8117292; doi:10.1186/s12875-021-01441-9)
Supplement: Supplementary file 1 — Additional file 1 [file 12875_2021_1441_MOESM1_ESM.docx]

**Additional file 1** CReDECI 2 checklist

| **Item** | | **Reported on page or in publication** |
| --- | --- | --- |
| **First stage: Development** | |  |
| 1 | Description of the intervention’s underlying theoretical basis | pp. 3-5; pp. 10-14, p. 18-19 |
| 2 | Description of all intervention components, including the reasons for their selection as well as their aims / essential functions | pp. 21-22 |
| 3 | Illustration of any intended interactions between different components | pp. 21-22 |
| 4 | Description and consideration of the context’s characteristics in intervention modelling | Figure 4 |
| **Second stage: Feasibility and piloting** | |  |
| 5 | Description of the pilot test and its impact on the definite intervention | Not applicable |
| **Third stage: Evaluation** | |  |
| 6 | Description of the control condition (comparator) and reasons for the selection | Not applicable |
| 7 | Description of the strategy for delivering the intervention within the study context | Not applicable |
| 8 | Description of all materials or tools used delivery the intervention | Not applicable |
| 9 | Description of fidelity of the delivery process compared the study protocol | Not applicable |
| 10 | Description of a process evaluation and its underlying theoretical basis | Not applicable |
| 11 | Description of internal facilitators and barriers potentially influencing the delivery of the intervention as revealed by the process evaluation | Not applicable |
| 12 | Description of external conditions or factors occurring during the study which might have influenced the delivery of the intervention or mode of action ( how it works) | Not applicable |
| 13 | Description of costs or required resources for the delivery of the intervention | Not applicable |
