# Supplementary material for: Development of a complex intervention to improve mobility and participation of older people with vertigo, dizziness and balance disorders in primary care: a mixed methods study
Source: BMC Fam Pract. 2021 May 12;22:89. doi: 10.1186/s12875-021-01441-9 (PMC8117292; doi:10.1186/s12875-021-01441-9)
Supplement: Supplementary file 2 — Additional file 2 [file 12875_2021_1441_MOESM2_ESM.docx]

**Additional file 2** Interview guides for individual interviews

**a) with patients**

**Introduction**

Thank you for agreeing to take part in our survey.

My name is [name of the research associate], I am a research associate in the research project. My colleague [name of the second person present] will take the minutes.

**Data protection**

As we have already informed you in writing, our conversation will be audio-recorded so that we do not miss any of your statements and our evaluation can take place afterwards. For this purpose, we have sent you a data protection declaration in advance that your statements will be processed anonymously and that you can interrupt or cancel the interview at any time.

**Setting**

In the following 15 minutes we want to ask about your experiences and needs regarding your medical care. Please answer freely, there are no wrong answers.

Would you like to hear the aim of our research project again before we start?

[If so:] The aim of our research project is to improve the care of patients with vertigo, dizziness and balance disorders. In order to achieve this, we want to develop a schedule that should help those involved in the care to select the optimal treatment. We conduct interviews with patients because it is important for us to get to know the situation of those affected better and to incorporate these experiences into our project.

Do you still have questions about the procedure? [Break]

You are welcome to ask questions at any time!

Then I would like to start the interview now.

| **Content aspects / dimension** | **Key questions** | **Maintenance questions** | **Memos** | **Specific questions** |
| --- | --- | --- | --- | --- |
| **First contact experience** | You have agreed to take part in our study because you have vertigo, dizziness or impaired balance.  Please tell me how you noticed this? | Is there anything else?  Can you describe that in more detail?  Do you have an example that I can imagine more precisely? | - Complaints | - Dizziness or imbalance? - How long have you had these complaints? - Additional complaints? Headache, fall, unsteady gait, drowsiness? - How do your symptoms occur? (Coping with everyday life, during the day, household, personal hygiene, family, friends, meeting acquaintances, pursuing hobbies) |
|  |  |  | - Diagnosis known? | - Do you have any idea why you are dizzy? - Are there any other issues related to this? - Has the GP / specialist found anything else? |
| **Experience treatment** | What does the general practitioner / specialist advise you? | Was there anything else recommended to you, e.g. ... | - | - Physiotherapy, medication, lifestyle, diet ... |
|  |  |  |  | [If physiotherapy:]   - Did you get a prescription? - Who issued this? - How many treatments did you have? - Did you notice them? - Are they relieving your symptoms? - Was the number of appointments appropriate for you - Describe a typical session with the therapist. How was the last session? - Were you able to find a suitable therapist straight away? |
| **Care process** | You said that you had gone through [name steps: e.g. family doctor, specialist, physiotherapy].  How did you feel about this process? | How was that for you? | - | - Good success? - Bad success? |
| **Need** | You said that you had gone through [name steps: e.g. family doctor, specialist, physiotherapy]. What has helped you the most? |  | - Generally? - At the family doctor? - At the physiotherapist? | - Is there something that you still needed, that is, something that was missing? |
| **Wish** | Now we are almost at the last question: Imagine that you were queen / king and could decide everything. What would your ideal care look like for you in an ideal health system? | Would you like to say something overall that has not been taken into account so far? |  | Chronic course / sustainability:  Which offers in general?  Individual or group intervention? In a block or weekly?  What time expenditure? (Travel time / pick-up service |
| **Demographics** | How old are you? | | | |
| **Insurance type** | How are you insured? Public or private? | | | |

**End**

Then we are now at the end of the interview. We would like to thank you again for your participation.

**b) with health professionals (example of GPs)**

**Introduction**

Thank you for agreeing to take part in our survey.

My name is [name of the research associate], I am a research associate in the research project. My colleague [name of the second person present] will take the minutes.

**Data protection**

As we have already informed you in writing, our conversation will be audio-recorded so that we do not miss any of your statements and our evaluation can take place afterwards. For this purpose, we have sent you a data protection declaration in advance that your statements will be processed anonymously and that you can interrupt or cancel the interview at any time.

**Setting**

In the following 15 minutes we want to ask about your experiences and needs as a family doctor in outpatient care on the subject. Answer freely, there are no wrong answers.

Would you like to hear the aim of our research project again before we start?

[If so:] The aim of our research project is to improve the care of patients with vertigo, dizziness and balance disorders. To achieve this, we want to develop a treatment pathway. We will conduct the interview with you so that general practitioners who treat patients with vertigo, dizziness and balance disorders can also have their say about what would be important.

Do you still have questions about the procedure? [Break]

You are welcome to ask questions at any time!

Then I would like to start the interview now.

| **Content aspects / dimension** | **Key questions** | **Maintenance questions** | **Memos** | **Specific questions** |
| --- | --- | --- | --- | --- |
| **Experience patients with dizziness or impaired balance** | You have agreed to take part in our study because you are treating patients with dizziness or impaired balance.  Please tell me which patients do you meet exactly? | Is there anything else?  Can you describe that in more detail?  Do you have an example that I can imagine more precisely?  Would you like to say something overall that has not been taken into account so far? | More than 65 years | - Frequency of dizziness in patients? - Frequency of patients with balance disorders? - Do patients come primarily because of dizziness or balance disorders? - Which causes can you observe most often? Medication influence? - Do you make a random diagnosis / as an accompanying symptom? - [If so:] From what? |
| **Self-experience as a family doctor** | As a general practitioner, what diagnostic options do you use to examine patients with the main symptoms of dizziness and balance disorders? |  | Referral to neurologists, ENT, specialist centers? Cooperation to diagnosis (GP as coordinator? | - Which do you use most often? - • Do patients come from other specialists or do you refer to others? |
| **Experience treatment approaches** | What treatment approaches have you had good experiences with in this context? |  | (E.g. do not necessarily name, if necessary: the literature gives information on ...)  Physiotherapy, medication, alternative methods, diet, lifestyle recommendations? | - [if no PT mentioned:]   Are you considering physical therapy as a treatment option for people with dizziness and balance disorders |
|  |  |  |  | - What approaches do you think physiotherapy have to treat patients with dizziness or balance disorders? |
|  |  |  | Cervical spine syndrome?  Orthopedic: KG, MT, KGG  Neurological: KG-CNS (CN2: Parkinson's, MS ...)  Special: KG + ÜB: SO3 (vertigo of various origins)  Long-term need: Parkinson's (Hoehn Yahr 5)  Received and read therapy report | - [if PT is mentioned:]   How often a month do you as a family doctor currently write a prescription for physiotherapy for patients with dizziness and balance disorders?   - For which indication exactly? - What do you choose from the catalog of remedies? - Which factors influence your decision to write a follow-up prescription? - Do you request a therapy report from the physiotherapist? Which aspects influence whether you do this? |
|  |  |  | - | - Do you check the medication schedule for the dizziness side effect? |
| **Need** | Is there anything that you think is doing particularly well in the current care of patients with dizziness and balance disorders? |  | Diagnosis?  Treatment?  Interdisciplinary communication? | - What are the problems in practice? - From the insurances? |
|  | Is there anything that in your opinion is not yet working so well in the supply process for this target group? |  |  |  |
| **Wish** | Now we are at the last question. Before that, a somewhat special question, the so-called king question. Imagine if you were queen / king and could decide everything. What would the ideal care look like for you in an ideal health system? |  | - | - |

| **Demographics** | How old are you? |
| --- | --- |
| **Work experience** | How long have you been working in your job? |

**End**

Then we are now at the end of the interview. We would like to thank you again for your participation.
