# Supplementary material for: Development of a complex intervention to improve mobility and participation of older people with vertigo, dizziness and balance disorders in primary care: a mixed methods study
Source: BMC Fam Pract. 2021 May 12;22:89. doi: 10.1186/s12875-021-01441-9 (PMC8117292; doi:10.1186/s12875-021-01441-9)
Supplement: Supplementary file 3 — Additional file 3 [file 12875_2021_1441_MOESM3_ESM.docx]

**Additional file 3** Worksheets to model behaviour change

Steps, activities and derived solutions guiding through development of behaviour change

| **Steps** | | **Activities** | **Solution** | | |
| --- | --- | --- | --- | --- | --- |
| 1 | Define the problem in behavioural terms | What behaviour problem, who is involved and where does it occur? | | - HPs apply no consistent or standardized EBP to patients with VDB |  |
| 2 | Select and specify target behaviour | Who needs to do what differently, when, where, how often and with whom? | | - HPs apply EBP in the form of our CPW to VDB patients in primary care / ambulant setting |  |
| 3 | Identify what needs to change (COM-B and TDF) | What needs to happen for the target behaviour to occur? Is there a need for change? | | - Behavioural diagnosis:   Psychological capability, physical opportunity, social opportunity, reflective and automatic motivation need to change in order for the target behaviour |  |
| 4 | Identify intervention functions | Use given linkages. Does the intervention function meet the APEASE criteria? | | - Intervention functions:   Education, incentivisation, training, environmental restructuring and enablement |  |
| 5 | Identify policy strategies | Complete this step only when you have access to policy levers. | | - Not applicable |  |
| 6 | Identify behaviour change techniques and draft an intervention strategy | Use given examples. Does the BCTs meet the APEASE criteria? | | - BCTs: - educational training contenting prompts / cues, - demonstration of pretended skills and do-it-yourself-elements with feedback - accompanying information or instruction manuals - social support by mentoring during first phase of applying - accreditation points for educational training or case rates |  |
| 7 | Identify modes of delivery | Use given examples. Does the mode of delivery meet the APEASE criteria? | | - Mode of delivery: - Education: group setting face-to-face - Phone helpline: individual-level in distance. |  |
| APEASE = Affordability, Practicability, Effectiveness, Acceptability, Side-effects / safety, Equity; BCTs = Behaviour change techniques; COM-B = Capability, opportunity, motivation and behaviour model; CPW = Care pathway; EBP = Evidence-based practice; HPs = Health professionals; TDF = Theoretical domains framework. | | | | |  |

1. Definition of problem behaviour

HPs apply no consistent or standardized EBP to patients with VDB.

1. Definition of target behaviour

HPs apply EBP in the form of our CPW to VDB patients in primary care / ambulant setting.

1. Fulfilled COM-B and TDF assessment guiding to behavioural diagnosis with additionally assigned CFIR constructs identified at our expert workshop

| **COM-B** | **TDF** | **Additional CFIR** | **What needs to happen for the target behaviour to occur** | **Is there a need for change?** |
| --- | --- | --- | --- | --- |
| **Physical**  **Capability** | Physical skills | - | HPs have the physical skills to apply evidence-based practice. | No change needed as HPs have this skills. |
| **Psychological**  **capability** | Knowledge | Knowledge about intervention | HPs understand why EBP is necessary in general and what is EBP in VDB patients. | Change needed to build or strengthen opinion of EBP and generate specific knowledge of HPs. |
|  | Cognitive and interpersonal skills | Individual Stage of Change; Reflecting and evaluating | HPs apply skills exactly for EBP and receive feedback. | Change needed to strengthen use of skills. |
|  | Memory, attention and decision processes | - | EBP is something HPs usually do and they need nothing to remember to do EBP in daily decision making. | No change needed because not relevant. |
|  | Behavioural regulation | - | HPs use material for self-monitoring. | No change needed because not relevant. |
| **Physical**  **opportunity** | Environmental context and resources | Implementation Climate; Available resources | HPs are able to apply EBP because of owning devices or manuals and practice has resources to implement new intervention. | Change needed to apply EBP in practice because of getting information and adequate devices or manuals. |
| **Social**  **opportunity** | Social influences | Organizational incentives or rewards; Leadership engagement | HPs are able to apply EBP and become a known specialist. Leadership empowers this. | Change needed to become a specialist by professional social support |
| **Reflective**  **Motivation** | Professional / social role and identity | - | HPs think that applying EBP is best practice. | No change needed, HPs should have this aim and EBP is not in conflict with role. |
|  | Beliefs about capabilities | Goals and feedback | HPs belief in their capabilities. | Change needed because it is easier to build self-confidence if educational training and/or mentoring. |
|  | Optimism | - | HPs are confident in applying EBP. | Change needed, that HPs see the benefit of applying the CPW. |
|  | Beliefs about consequences | Beliefs about intervention | HPs think they can help patients or treat better. | Change needed, that HPs think they act more effective using the CPW. |
|  | Intentions | Tension for change | Decision to do EBP in general was made by law but in every day practice by HPs themselves. | No change needed because not relevant. |
|  | Goals | Goals and feedback; Planning | HPs want to do EBP because they feel more certain with VDB patients. | No change needed because HPs yet feel uncertain with VDB patients. |
| **Automatic**  **Motivation** | Reinforcement | Organizational incentives or rewards; Reflecting and evaluating | HPs want to occur as a known specialist. | Change needed to organize reputation of HPs. |
|  | Emotion | Individual Stage of Change | HPs have pleasure to apply new method. | Change needed because HPs might have stress when applying new method. |
| **🡪Behavioural diagnosis** | Psychological capability, physical opportunity, social opportunity, reflective and automatic motivation need to change in order for the target behaviour. | | | |

1. Identified intervention functions

| **Intervention function /**  **COM-B and TDF component** | **Education**  *🡪Identify and prepare champions*  *🡪Conduct educational meetings*  *🡪Identify early adopters* | **Persuasion** | **Incentivisation**  *🡪Alter incentive / allowance structures*  *🡪* *Access new funding* | **Coercion** | **Training**  *🡪Identify and prepare champions*  *🡪Conduct educational meetings*  *🡪Identify early adopters* | **Restriction** | **Environmental restructuring** | **Modelling** | **Enablement**  *🡪Identify and prepare champions*  *🡪Identify early adopters* |
| --- | --- | --- | --- | --- | --- | --- | --- | --- | --- |
| **Physical capability** |  |  |  |  | / |  |  |  | / |
| **Psychological capability** | Educate about EBP and generate specific knowledge. |  |  |  | Strengthen skills how to use EBP exactly. |  |  |  | Enable development of skills. |
| **Physical opportunity** |  |  |  |  | Train HPs in spreaded information and materials | / | Structure environment by spreading information or manuals |  | / |
| **Social opportunity** |  |  |  |  |  | / | Structure social environment by promoting utilization of mentoring support. | / | Enable development of experience by social support by mentoring |
| **Reflective Motivation** | Build self-confidence by educational training | | / | / |  |  | . |  |  |
| **Automatic Motivation** |  | Build positive opinion about EBP | Organize reputation. | / | Train new method and avoid stress when apply-ing |  | Build self-confidence by mentoring | / | / |

1. Judgement of selected intervention function according to the APEASE criteria

| **Candidate**  **intervention**  **function** | **Does the intervention function meet the APEASE criteria?** |
| --- | --- |
| **Education** | Yes. Training materials are possible to develop but budget has to be redesignated for a trainer; training workshop has to be short and time point matched to HPs` availability; Effects are estimated high when professional trainer; workshops are established in HPs. |
| **Persuasion** | No. Unlike to be effective as most HPs have a positive opinion to EBP |
| **Incentivisation** | Yes. Organize reputation is possible to do in the way of a practice list or seal of quality. |
| **Training** | Yes. s. Education |
| **Env. restructuring** | Yes. Information materials are possible to develop and a mentor is possible to recruit out of collaborators |
| **Enablement** | Yes. s. Education and Environmental restructuring |
| **🡪Selected**  **intervention functions** | Education, Incentivisation, Training, Environmental restructuring and Enablement |

1. Intervention functions leading to behaviour change techniques and intervention strategy

| **Intervention function** | **Individual BCT** | **COM-B** | **TDF** | **TDF BCT** | **Does the BCT meet the APEASE criteria?** | **🡪Intervention strategy** |
| --- | --- | --- | --- | --- | --- | --- |
| Education | Prompts / cues | Psychological capability | Knowledge | / | Yes. Prompts / cues can be given by a trainer who is a medical specialist to educate about EBP and generate specific knowledge | To apply EBP by HPs first need an educational training contenting prompts / cues, demonstration of pretended skills and do-it-yourself-elements with feedback.  Second, accompanying information or instruction manuals should be given to HPs as well as a social support by mentoring during first phase of applying.  At least, a form of material incentive like accreditation points for educational training or case rates seems useful for participation. |
|  | Feedback on behaviour | Reflective Motivation |  | Feedback on behaviour | Yes. Do it yourself elements with feedback by trainer in educational workshop can build practical knowledge and build self-confidence. |  |
|  |  |  | Beliefs about capabilities | Verbal persuasion to boost self-efficacy | No. Verbal persuasion seems not to be practicable because of educational training by a medical specialist but not a coach. |  |
| Incentivisation | Social reward | Automatic motivation | Reinforcement | Social reward | Yes. Organizing social reward by giving seal of quality or material reward by accreditation points and case rates is possible to organize. |  |
|  | Material reward |  |  | Material reward |  |  |
| Training | Demonstration of the behaviour | Psychological capability | Skills | Behavioural practice | Yes. Pretended skills and exact apply can be demonstrated in workshop. |  |
|  | Instructions in how to perform the behaviour | Physical opportunity | Environmental context and resources | Restructuring the physical environment | Yes. Giving information or instruction manuals can be used from collaborating partners or developed. |  |
| Environmental restructuring | Adding objects to the environment | Social opportunity | Social influences | Social support (practical) | Yes. Social support by mentoring can be asked by a trainer. |  |
|  | Restructuring the physical environment | Physical opportunity | Environmental context and resources | Restructuring the physical environment | Yes. Giving information or instruction manuals can be used from collaborating partners or developed. |  |
| Enablement | Social support (practical) | Social opportunity | Social influences | Social support (practical) | Yes. Social support by mentoring can be asked by a trainer. |  |

1. Identification of mode of delivery

| **Mode of delivery** | | | | **Does the mode of delivery meet the APEASE criteria?** |
| --- | --- | --- | --- | --- |
| Face-to-face | Individual | | | No. Not practicable and not responsible to resources. |
|  | Group | | | Yes. For educational training useful. |
| Distance | Population-level | Broadcast media | TV | No. Tutorials of educational training would be possible, but missing effects like feedback by trainer. |
|  |  |  | Radio | No. |
|  |  | Outdoor media | Billboard | No. |
|  |  |  | Poster | No. |
|  |  | Print media | Newspaper | No. |
|  |  |  | Leaflet | No. |
|  |  | Digital media | Internet | No. Tutorials of educational training would be possible, but missing effects like feedback by trainer. |
|  |  |  | Mobile phone app | No. Tutorials of educational training would be possible, but missing effects like feedback by trainer. |
|  | Individual-level | Phone | Phone helpline | Yes. Help line serves social support. |
|  |  |  | Mobile phone text | No. |
|  |  | Individually accessed computer program | | No. |
